# In Vitro Evaluation of the Inhibitory Potential of Pharmaceutical Excipients on Human Carboxylesterase 1A and 2

**DOI:** 10.1371/journal.pone.0093819

**Published:** 2014-04-03

**Authors:** Chengliang Zhang, Yanjiao Xu, Qiaoni Zhong, Xiping Li, Ping Gao, Chengyang Feng, Qian Chu, Yuan Chen, Dong Liu

**Affiliations:** 1 Department of Pharmacy, Tongji hospital, Tongji medical school, Huazhong University of Science and Technology, Wuhan, Hubei, China; 2 Hubei Pharmaceutical Industry Research Institute Co. Ltd., Wuhan, Hubei, China; 3 Department of Pharmacy, Wuhan Children's Hospital, Wuhan, Hubei, China; 4 Department of Oncology, Tongji hospital, Tongji medical school, Huazhong University of Science and Technology, Wuhan, Hubei, China; University of Parma, Italy

## Abstract

Two major forms of human carboxylesterase (CES), CES1A and CES2, dominate the pharmacokinetics of most prodrugs such as imidapril and irinotecan (CPT-11). Excipients, largely used as insert vehicles in formulation, have been recently reported to affect drug enzyme activity. The influence of excipients on the activity of CES remains undefined. In this study, the inhibitory effects of 25 excipients on the activities of CES1A1 and CES2 were evaluated. Imidapril and CPT-11 were used as substrates and cultured with liver microsomes *in vitro*. Imidapril hydrolase activities of recombinant CES1A1 and human liver microsomes (HLM) were strongly inhibited by sodium lauryl sulphate (SLS) and polyoxyl 40 hydrogenated castor oil (RH40) [Inhibition constant (K_i_) = 0.04±0.01 μg/ml and 0.20±0.09 μg/ml for CES1A1, and 0.12±0.03 μg/ml and 0.76±0.33 μg/ml, respectively, for HLM]. The enzyme hydrolase activity of recombinant CES2 was substantially inhibited by Tween 20 and polyoxyl 35 castor oil (EL35) (K_i_ = 0.93±0.36 μg/ml and 4.4±1.24 μg/ml, respectively). Thus, these results demonstrate that surfactants such as SLS, RH40, Tween 20 and EL35 may attenuate the CES activity; such inhibition should be taken into consideration during drug administration.

## Introduction

Pharmaceutical excipients, defined as the indispensable constituents of pharmaceutical formulations, are used to guarantee appropriate physicochemical and biopharmaceutical properties. Given their varied functions and properties, pharmaceutical excipients act as diluents/fillers, binders, disintegrants, lubricants, colouring agents and coating agents [1]. These adjuvants are conventionally considered to be inert from a pharmacological point of view and ideally should not affect the intended therapeutic action of the active substance. The most commonly used excipients have been extensively employed in the pharmaceutical industry and are generally believed to be devoided of biological effects. However, the safety of these vehicles remains to be completely understood and warrants further investigation. Several robust studies evaluating the suitability of excipients have identified many unexpected excipients-related toxicities. Tween 80 has been reported to augment the toxicity of drugs such as amiodarone, causing acute hepatotoxicity and also decreasing the glutathione levels [2]. Ingestion of a high dose of propylene glycol (PG) may cause cardiovascular and renal failure, central nervous system changes and hyperosmolality [3]. Formulations containing hydroxypropyl-beta-cyclodextrin (HP-β-CD) produced elevated transaminase (aspartate and alanine aminotransferase) levels in rats and mice and faecal changes (loose and soft stool) in large animals such as dogs and monkeys [4]. Certain solubilizing agents appear to alter the intestinal membrane barrier functions and cause damage to the intestinal epithelium [5]. With regard to the safety of the excipients, over the years, the US Food and Drug Administration (FDA) has published a list entitled ‘Inactive Ingredient Guide (IIG)’ (FDA, 2010) for excipients that have been approved and incorporated in the marketed products [6]. This guide is extremely helpful and provides a database of allowed excipients with the relevant maximum dosage level by route of administration or dosage form.

More recently, there are increasing concerns that excipients may have considerable physiological and physicochemical effects on drug metabolism enzymes as well as on transporting proteins, which can inevitably influence drug disposition [7]. There is some evidence in the literature that a number of excipients can reduce the function of P-glycoprotein (P-gp), resulting in increased intestinal absorption of P-gp substrates [8]–[14]. Polyethylene glycol (PEG)400, HP-β-CD, Solutol HS 15 (SOL), and Cremophor EL (CrEL) can considerably modulate the function of the organic anion-transporting polypeptides (OATPs) and the Na^+^/taurocholate cotransporting polypeptide (NTCP) [15]. Another major mechanism by which excipients modulate drug process *in vivo* is alteration of the activities of drug metabolism enzymes. For example, Bravo Gonzalez reported that the metabolism of midazolam, a substrate of CYP3A4, could be affected by polyoxyl 35 castor oil (EL35), Tween 80, PEG400 and SOL *in vitro* [16]. Another study conducted by Ren also demonstrated that EL35, Tween 80 and PEG 400 inhibited the activity of CYP3A4, and thus influenced the metabolism of the active drug [17]. Buggins et al reviewed the reported effects of commonly used pharmaceutical excipients on drug disposition, and confirmed that such information was useful in study design and evaluating data from pharmacokinetic experiments [1].

Carboxylesterases (CESs) are members of the α/β hydrolase fold family and play an important role in biotransformation of a variety of ester-containing drugs, pesticides, and prodrugs such as angiotensin-converting enzyme inhibitors (temocapril, cilazapril and imidapril), anti-tumour drugs [irinotecan (CPT-11) and capecitabine] and narcotics (cocaine, heroin and meperidine) [18]. CESs tend to locate in the epithelia that are likely to be exposed to xenobiotics, and the plastic nature of their active sites can accommodate substrates with varied structures [19]. Human CESs comprise a multigene family, and CES1A and CES2 families are the two major isoforms of CESs. CES1A is predominantly expressed in the liver and the lung, whereas CES2 is expressed in the gastrointestinal tract and the liver [20]. Human CES1A and CES2 govern the pharmacokinetic behaviours of most prodrugs, and the inhibitory effect on their activities by chemicals is vital for the potency of drugs. Fukami T *et al* examined the inhibitory effects of various antidiabetic and antihyperlipidemic drugs on human CES enzyme activity, and observed that simvastatin [inhibition constant (K_i_) = 0.11 μM], troglitazone (K_i_ = 0.62 μM) and fenofibrate (K_i_ = 0.04 μM) inhibited CES1A1 and CES2 enzyme activities [21]. Our previous study investigated the inhibitory effects of 17 antihypertensive drugs on the CES activity and found that diltiazem, verapamil, nitrendipine and telmisartan could attenuate the drug efficacy of catalysed prodrugs by altering the activities of CES1A1 and CES2 [22]. So far, there were hardly any systematic studies assessing the inhibitory potential of excipients on CES activity in humans have been performed or published to date. Thereby, we herein investigated the inhibitory effects of 25 common pharmaceutical excipients on the activities of CES1A and CES2 *in vitro*.

## Materials and Methods

### Materials

Imidapril and imidaprilat were purchased from Sigma (St. Louis, MO). CPT-11, SN-38, *p*-nitrophenyl acetate and *p*-nitrophenol were purchased from Toronto Research Chemicals Inc (North York, ON, Canada). Cetirizine (purity >99.7%) and camptothecine were purchased from the National Institute for the Control of Pharmaceutical and Biological Product (Beijing, China). PG, glycerin and lactose were purchased from Wuhan Chemicals Ltd. (Wuhan, China). PEG 200, PEG 400, PEG 4000, PEG 6000, microcrystalline cellulose (MCC) and carboxymethylcellulose sodium (CMC-Na) were purchased from Dow Chemicals Ltd. (Midland, MI, USA). Poloxamer 188 (F68), sodium lauryl sulphate (SLS), hydroxypropylcellulose (HPC), povidone (PVP) and sodium alginate were purchased from BASF (Germany). Lecithin, oleic acid and mannitol were purchased from Jiade Pharmaceutical Co. (Beijing, China). Triton X-100, Polyoxyl 35 castor oil (EL35), polyoxyl 40 hydrogenated castor oil (RH40), Tween 20 and Tween 80 were purchased from Cognis UK Ltd. (Southampton, Hampshire, UK). Polyoxyl 40 stearate (S40) was purchased from Adina Chemicals Ltd. (Tunbridge Wells, Kent, UK). Sodium bisulphite (NaHSO_3_) and ascorbic acid (Vit.C) were purchased from Hubei Pharmaceutical Corporation (Wuhan, Hubei, China). Pooled human liver microsomes (HLM) were purchased from BD Gentest (Woburn, MA) and pooled human jejunum microsomes (HJMs) were purchased from Tissue Transformation Technologies (Edison, NJ). Human CES1A1 and human CES2 expressed in baculovirus-insect cells were obtained from BD Gentest (Woburn, MA). All other chemicals and solvents were of analytical or HPLC grade.

### Imidaprilat Formation

Formation of imidaprilat from imidapril was determined according to the method described previously with a slight modification [23]. The incubation mixture (200 μl of total volume), including the microsomes, 100 mM Tris-HCl buffer (pH 7.4) and inhibitors, was preincubated at 37°C for 2 min. The reaction was initiated by the addition of imidapril (100 μM), followed by incubation of the mixture at 37°C for 15 min. The reaction was terminated by adding 200 μl of ice-cold ethyl acetate and the mixture was next dried under N_2_. Cetirizine was added as an internal standard. After centrifugation at 15000 g for 5 min, imidaprilat in the supernatant (10 μl) was quantified employing a liquid chromatography-tandem mass spectrometry system. Diamonsil C_18_ (5 μm,150×2.1 mm) was used as the analytical column, with acetonitrile-0.1% (v/v) formic acid (1∶2, v/v) as the mobile phase at a flow rate of 0.3 mL·min^−1^. The calibration curve of imidaprilat in the incubation mixture was linear over a concentration range of 5–1000 ng·ml^−1^ (r = 0.9991). Interday and intraday coefficients of variation of the different quality control samples were both less than 10%.

### SN-38 Formation

Formation of SN-38 from CPT-11 was determined according to the method described previously with a slight modification [24], [25]. A typical incubation mixture (200 μl of total volume), including the microsomes, 100 mM phosphate buffer (pH 7.4) and inhibitors, was preincubated at 37°C for 2 min. The reaction was initiated by the addition of CPT-11 (5 μM), following which the mixture was incubated at 37°C for 10 min. The reaction was terminated by adding 200 μl of ice-cold methanol. Camptothecine was included as an internal standard. After centrifugation at 15,000 g for 10 min, supernatant (10 μl) was analyzed by high performance liquid chromatography. CPT-11 and SN-38 were separated on a Hypersil ODS C_18_ (4.6 mm×250 mm, 5 μm) column using the mobile phase of 0.05 mol·L^−1^ phosphate buffer-methanol (50∶50, v/v) containing 0.25‰ triethylamine (pH adjusted to 3.0 with phosphate buffer) and monitored at excitation and emission wavelengths of 380 nm and 550 nm, respectively. The standard curve of SN-38 in the incubation mixture ranged from 2 to 400 ng·ml^−1^ with a correlation coefficient (r = 0.9975). Interday and intraday coefficients of variation of the quality controls were in the range of 5.6–11.2%.

### *p*-Nitrophenyl Acetate Hydrolase Activity

*p*-Nitrophenyl acetate hydrolase activity was determined according to the methods described previously [26].

### Inhibition Analysis of CES Enzyme Activitiy

The inhibitory effects of 25 excipients on imidapril and CPT-11 hydrolase activities were investigated. These excipients were classified into 6 groups based on their chemical and physical characteristics: (1) Adhesives: HPC, PVP, CMC-Na, PEG4000, PEG6000 and sodium alginate; (2) Fillers: lactose, MCC and mannitol; (3) Co-solvents: PG, glycerin, PEG200 and PEG400; (4) Surfactants: Tween 20, Tween 80, SLS, S40, F68, Triton X-100, RH40 and EL35; (5) Absorption enhancers: lecithin and oleic acid; (6)Antioxidants: Vit.C and NaHSO_3_. Lecithin and oleic acid were dissolved in DMSO, whereas the rest of the excipients were dissolved in distilled water. These excipients were added to the incubation mixtures described above to investigate their inhibitory effects on the imidaprilat and SN-38 formations. All data were analysed using the mean of duplicate determinations.

For screening the inhibitory effects, the enzyme activities at 100 μM imidapril and 5 μM CPT-11 were examined [27]. The concentrations of the tested excipients were 500 μM, except F68, EL35, RH40, lecithin, PVP and sodium alginate that were used a concentration of 100 μg/ml. For determination of the K_i_ values of the imidapril hydrolase activity, imidapril concentrations ranging from 25 to 200 μM were used. The concentrations of the inhibitors for the imidapril hydrolase activity ranged as follows: SLS, 0.03–0.16 μg/ml for recombinant CES1 and 0.08–0.32 μg/ml for HLM, and RH40, 0.15–2 μg/ml for recombinant CES1 and 0.5–5 μg/ml for HLM. The protein concentrations of HLM and CES1A1 were 0.12 mg/ml and 0.38 mg/ml, respectively.

For determination of the Ki values, the concentrations of CPT-11 ranged from 2 to 20 μM for recombinant CES2 and 5 to 50 μM for HLM and HJM, respectively. The concentrations of the inhibitors for the CPT-11 hydrolase activity ranged as follows: Tween 20, 0.5–3 μg/ml for recombinant CES2, 2–10 μg/ml for HLM and 1–8 μg/ml for HJM; EL35, 3–15 μg/ml for recombinant CES2, 10–40 μg/ml for HLM and 8–30 μg/ml for HJM. The protein concentrations of recombinant CES2, HLM and HJM were 0.35 mg/ml, 0.05 mg/ml and 0.13 mg/ml, respectively.

To compare the inhibitory effects, the enzyme activities at 150 μM *p*-nitrophenyl acetate were examined. The concentrations of the tested excipients were 500 μM, except that F68, EL35, RH40, lecithin, PVP and Sodium alginate were 100 μg/ml. To determine the inhibitor concentration that caused 50% inhibition (IC_50_), the *p*-nitrophenyl acetate hydrolase activities by recombinant CES1A1 and CES2 at 150 μM were examined in the presence of the inhibitors. The concentration range of inhibitors was as follows: SLS, 0.02–0.5 μg/ml, RH40, 0.1–5 μg/ml, Tween 20, 0.5–15 μg/ml, and EL 35, 3–40 μg/ml.

The K_i_, K_m_ and V_max_ values and inhibition types were determined by fitting the kinetic data to a competitive, noncompetitive, uncompetitive or mixed inhibition model by the Enzyme Kinetics Module using SigmaPlot 12.0 (Systat Software, Inc., San Jose, CA). The K_i_, K_m_ and V_max_ values represent the mean ± S.E.

## Results

### Inhibitory Effects of 25 Excipients on Imidapril Hydrolase Activities of Recombinant Human CES1A1

**Figure 1:**
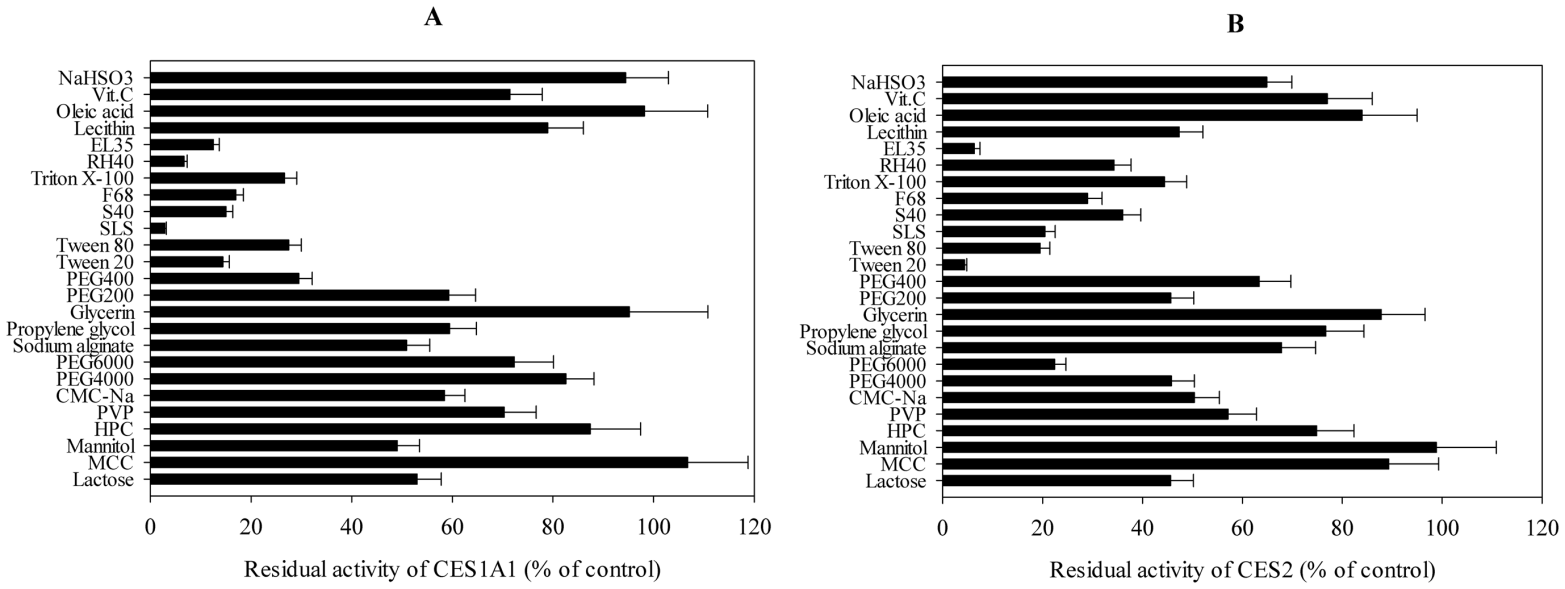
Inhibitory effects of excipients on CES1A1 and CES2. Inhibitory effects of 25 excipients on imidapril hydrolase activity of recombinant CES1A1 (A) and on CPT-11 hydrolase activity of recombinant CES2 (B). The concentrations of imidapril and CPT-11 were 100 μM and 5 μM, and the concentrations of the tested excipients were 500 μM except that F68, EL35, RH40, lecithin, PVP and Sodium alginate were 100 μg/ml. Each column represents the mean ± SD. The control activity was 1.83 nmol/min/mg and 13.7 pmol/min/mg for CES1A1 and CES2, respectively.

The inhibitory effects of the 25 excipients on the imidapril hydrolase activity of human recombinant CES1A1 were investigated (Fig.1A). DMSO (0.1%) inhibited the imidapril hydrolase activity of CES1A1 by 0.92%. The inhibition rates of the excipients dissolved in DMSO were calculated as the percentages of the tested activity compared with that of the control one after eliminating the impact of 0.1% DMSO. Imidaprilat formation catalyzed by CES1A1 was strongly inhibited by SLS (percentage of control: 2.9%), RH40 (6.7%), S40 (15%), EL35 (12.5%) and Tween 20 (14.4%). The activity of recombinant CES1A1 was moderately inhibited by PEG 400 and Tween 80 (20%–50% of the control), whereas all the remaining excipients inhibited less than 50% of the imidapril hydrolase activity.

Inhibitory Effects of 25 Excipients on CPT-11 Hydrolase Activities of Recombinant CES2.

To investigate the inhibitory effects of the drugs on human CES2 enzyme activity, the CPT-11 hydrolase activity that was suppressed by 0.1% DMSO by 1.12% was evaluated (Fig.1B). For excipients that were dissolved in DMSO, the inhibition rates of the excipients were calculated as the percentages of the experimental activity relative to that of the control one. CPT-11 hydrolase activity of recombinant CES2 was dramatically inhibited by Tween 20 (percentage of control: 4.38%) and EL 35 (6.34%), and moderately inhibited by F68 and PEG 6000 (20%–50% of the control). The others exhibited weak inhibition (>50% of the control).

### Inhibition Constant and Inhibition Patterns of Imidapril Hydrolase Activities of Recombinant Human CES1A1 and HLM

**Figure 2:**
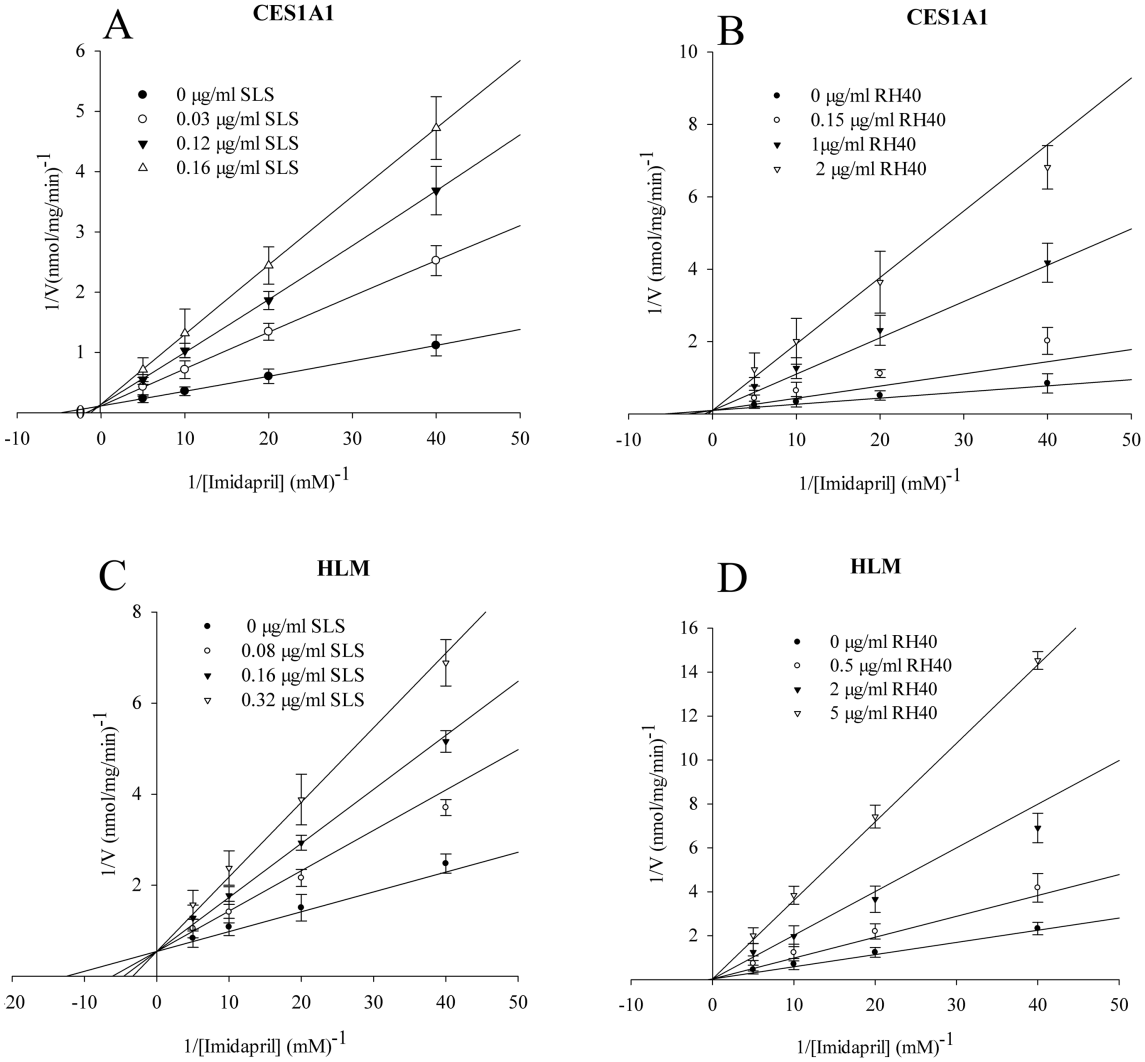
Inhibition constant (*K*_i_) and inhibition patterns for CES1A1 and HLM. Inhibitory effects of SLS (A and C) and RH40 (B and D) on the imidapril hydrolase activities of recombinant CES1A1 (A and B) and HLM (C and D). Each data point represents the mean ± SD. The *K*_i_ value represents the mean ±SE.

The Ki values and inhibition patterns of SLS and RH40 that strongly inhibited the imidapril hydrolase activities of recombinant CES1A1 and HLM were determined and the representative Lineweaver-Burk plots are illustrated in Fig.2. The Ki values of SLS and RH40 for recombinant CES1A1 were 0.04±0.01 μg/ml and 0.20±0.09 μg/ml, both with competitive-type inhibition. In contrast, the Ki values of SLS and RH40 for HLM were 0.12±0.03 μg/ml and 0.76±0.33 μg/ml, and the inhibition types were both competitive-type.

### 3.4. Inhibition Constant and Inhibition Patterns of CPT-11 Hydrolase Activities of Recombinant Human CES2, HLM, and HJM

**Figure 3:**
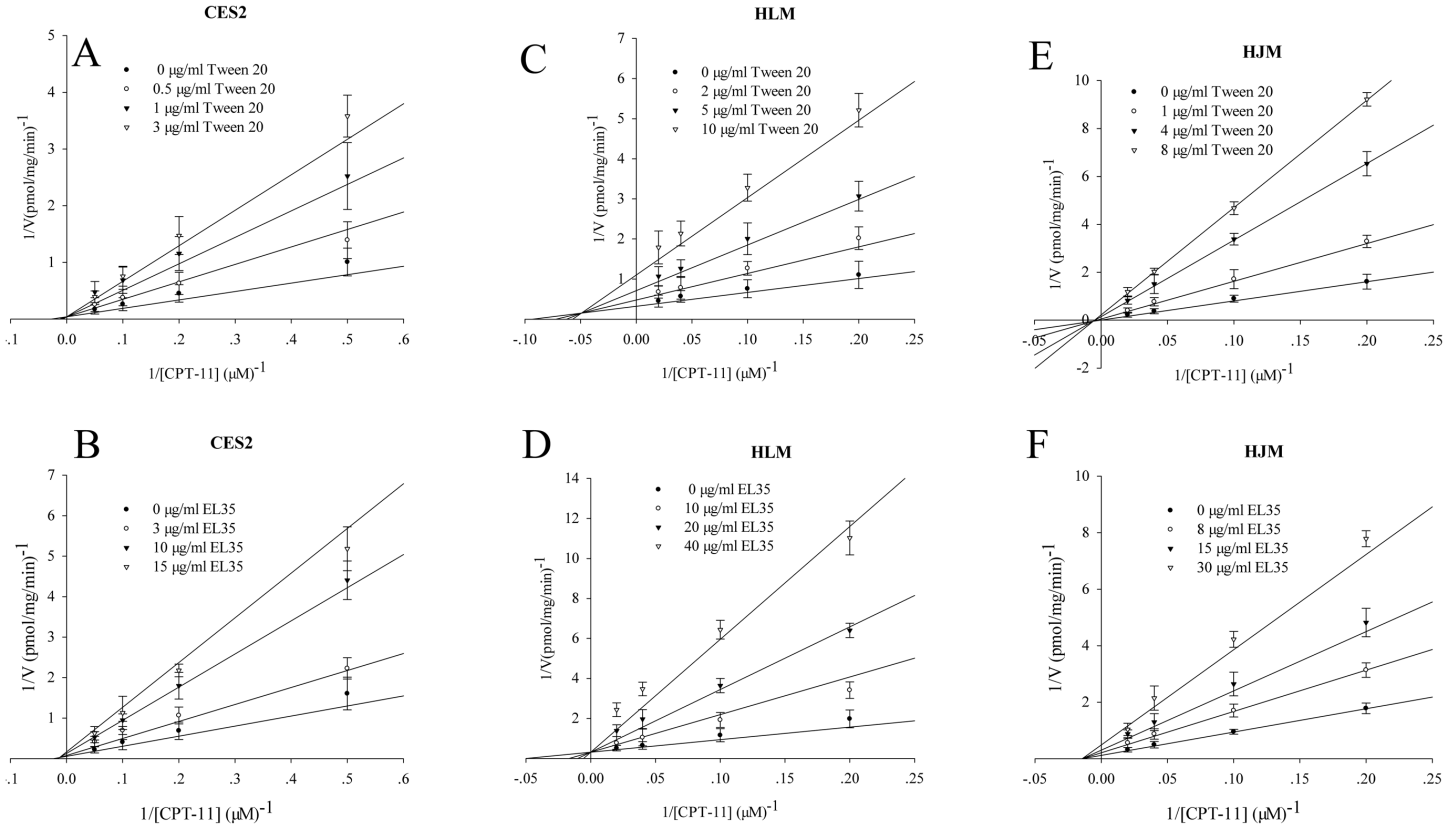
Inhibition constant (*K*_i_) and inhibition patterns for CES2, HLM and HJM. Inhibitory effects of Tween 20 (A, C and E) and EL35 (B, D and F) on the imidapril hydrolase activities of recombinant CES2 (A and B), HLM (C and D) and HJM (E and F). Each data point represents the mean ± SD. The *K*_i_ value represents the mean ±SE.

The Ki values and inhibition patterns of Tween 20 and EL35 that resulted in robust inhibition of CPT-11 hydrolase activities of recombinant CES2, HLM and HJM were determined. Representative Lineweaver-Burk plots are shown in Fig.3. The Km values for CPT-11 hydrolysis were similar for recombinant CES, HLM and HJM. The K_i_ values of Tween 20 and EL35 for recombinant CES2 were 0.93±0.36 μg/ml and 4.40±1.24 μg/ml with competitive- and mixed-type inhibition, respectively. The Ki values of Tween 20 and EL35 for HJM were 1.20±0.33 μg/ml and 13.2±2.0 μg/ml with mixed- and noncompetitive-type inhibition, respectively, whereas the values for HLM were 2.2±0.55 μg/ml and 20.54±3.82 μg/ml, respectively, with mixed- and competitive-type inhibition, respectively.

### Inhibitory Effects of the 25 Excipients on *p*-Nitrophenyl Acetate Hydrolase Activities of Recombinant Human CES1A1 and CES2

**Figure 4:**
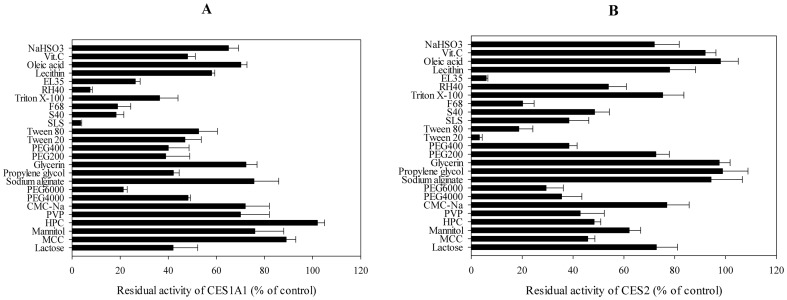
Inhibitory effects on *p*-nitrophenyl acetate hydrolase activities of recombinant CES1A1 (A) and CES2 (B). The activities were determined at 150 μM *p*-nitrophenyl acetate, and the concentrations of the tested excipients were 500 μM except that F68, EL35, RH40, lecithin, PVP and Sodium alginate were 100 μg/ml. Each data point represents the mean ± SD. The control activities by recombinant CES1A1 and CES2 were 2.62 nmol/min/mg and 35.8 pmol/min/mg, respectively.

To compare the inhibitory effects of the excipients on human CES1A1 and CES2 enzyme activities, *p*-nitrophenyl acetate hydrolase activities catalysed by both CES1A1 and CES2 were assessed (Fig. 4). DMSO (0.1%) inhibited the *p*-nitrophenyl acetate hydrolase activity by 0.78%. For excipients dissolved in DMSO, the inhibition rates were calculated as the percentage of the tested activity relative to that of the control one. We observed that SLS and RH40 remarkably inhibited (percentage of control: 3.6% and 7.5%, respectively) the *p*-nitrophenyl acetate hydrolase activity of CES1A1, but mildly inhibited that of CES2 (30–60% of the control). EL35 substantially suppressed (26.3% and 5.88%, respectively) the activities of CES1A1 and CES2. However, Tween 20 inhibited their activities differently (46.9% and 3.19% of the control, respectively). In addition, S40, F68 and PEG6000 led to modest inhibitions. The remaining excipients showed weak and diverse effects.

### IC_50_ Values of *p*-Nitrophenyl Acetate Hydrolase Activities of Recombinant Human CES1A1 and CES2

**Figure 5:**
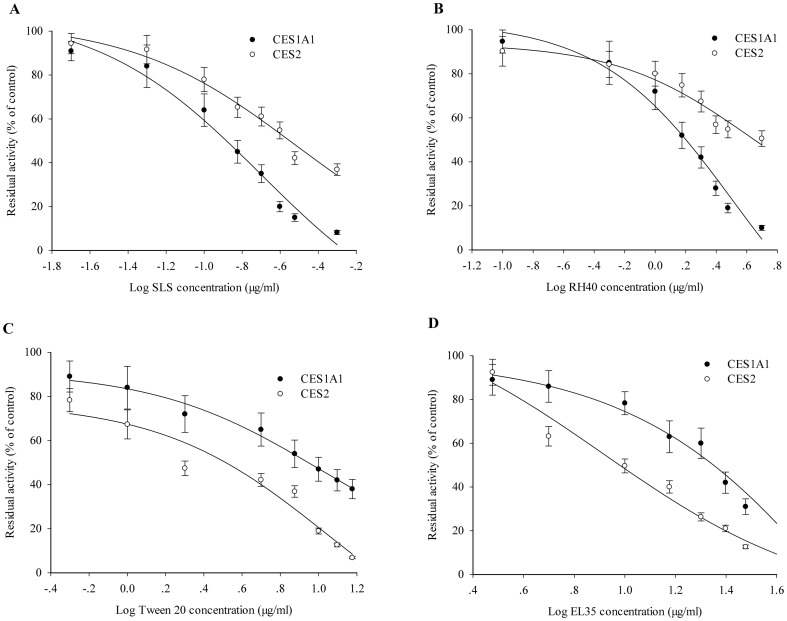
IC_50_ values of excipients for CES1A1 and CES2. IC_50_ values of SLS (A), RH40 (B), Tween 20 (C) and EL 35 (D) on the ***p***-nitrophenyl acetate hydrolase activities of recombinant CES1A1 and CES2. Microsomes were incubated with 150 μM *p*-nitrophenyl acetate in the presence of excipients of various concentrations. Activities were expressed as a percentage of the tested activity compared with the control. Each data point represents the mean ± SD. The control activities by recombinant CES1A1 and CES2 were 3.95 nmol/min/mg and 40.6 pmol/min/mg, respectively.

SLS and RH40 intensely inhibited the *p*-nitrophenyl acetate hydrolase activity of recombinant CES1A1 (Fig. 4A), whereas Tween 20 and EL35 apparently inhibited the activity of recombinant CES2 (Fig. 4B). To further compare the inhibitory effects of SLS, RH40, Tween 20 and EL35 on CES1A1 and CES2 enzyme activities, the IC_50_ values of *p*-nitrophenyl acetate hydrolase activity were determined (Fig. 5) using 150 μM substrate. The IC_50_ values of SLS and RH40 for recombinant CES1A1 were 0.20 μg/ml and 1.48 μg/ml, respectively, while those of Tween 20 and EL35 were 7.94 μg/ml and 12.59 μg/ml, respectively. On the other hand, the IC_50_ values of Tween 20 and EL35 for recombinant CES2 were 3.16 μg/ml and 8.22 μg/ml, respectively, and those of SLS and RH40 were 0.30 μg/ml and 4.05 μg/ml, respectively. Thus, the excipients demonstrated varied inhibitory effects on the activities of CES1A1 and CES2.

## Discussion

Pharmaceutical adjuvants are usually considered to be inert from a pharmacological point of view and are included in formulations to facilitate administration of the active ingredients. However, some adjuvants may pose safety concerns, and the lack of awareness of the possible risk from excipients may be an urgent issue for regulatory agencies, physicians and patients [28]. Excipients may alter enzyme activity thus altering the pharmacokinetics and efficacy of the drug. For example, previous studies have also reported the inhibitory effect of excipients on CYP enzymes. Tompkins found that HPMC, crospovidone (X-PVP), PEG-3350, PG, citric acid (CA), malic acid (MA) and polysorbate-80 (PS-80) decreased CYP3A4 protein expression in immortalised hepatocytes [29]. In addition, clinical significance was found as to the inhibitory effect of S40 on CYP2C9 and CYP2C19 [14]. Poloxamer 407 (P-407) has been reported to considerably elevate in plasma cholesterol and triglycerides in various animal models, including rats [30], [31], mice [32], [33] and rabbits [34]. An examination of the mechanism underlying P-407-induced hyperlipidemia demonstrated marked alterations in the activities of plasma hepatic lipase (EC 3.1.1.3) and lipoprotein lipase(EC 3.1.1.34), which belong to members of the α/β hydrolase fold family similar to CES (EC 3.1.1.1) [35].

Human CES1A and CES2, which are responsible for the biotransformation of both endogenous and exogenous compounds into polar products to be faecal elimination, eventually determine the drug efficacy. CES1 protein appears to be encoded by two separate, yet highly homologous CES1A1 and CES1A2 genes [36]. However, the levels of CES1A1 mRNA transcribed from the CES1A1 gene were substantially higher than those of CES1A2 mRNA transcribed from the CES1A2 gene [37]. Therefore, it is plausible that the level of CES1A1 mRNA rather than that of CES1A2 mRNA affects the level of mature protein and enzyme activity [38]. In the present study, we evaluated the inhibitory effects of 25 excipients on both CES1A1 and CES2 *in vitro* utilizing imidapril and CPT-11 as the representative substrates.

During the CES1-catalysed conversion of imidapril to imidaprilat, majority of (19 of 25) the tested excipients could inhibit the metabolic activity of CES1A1. SLS and RH40 strongly inhibited both HLM and recombinant CES1A1, and the K_i_ value of SLS for recombinant CES1A1 (0.04±0.01 μg/ml) was lower than that of RH40 (0.20±0.09 μg/ml). SLS and RH40 exhibited competitive-type inhibition for both HLM and CES1A1, suggesting that they may bind to the active site of CES1A1 which can accommodate substrates with varied structures [19]. Tween 20 and EL35 could inhibit the formation of SN-38 from CPT-11. In the present study, we observed that the K_i_ values for the inhibitory effects of Tween 20 on HLM, HJM and recombinant CES2 were all lower than the values for the inhibitory effects of EL35. In addition, the inhibitory patterns of Tween 20 and EL35 for HLM, HJM and recombinant CES2 were also different. According to our results, the inhibitory capacity of these excipients was determined by the diverse expression and structure of CES. First, CES1 mRNA expression is higher in the human liver than in the small intestine [20]. The concentration of human CES1 has been observed to be nearly 50-fold higher than that of CES2 in hepatic microsomes, according to quantitative immunoblotting data [39]. In contrast, human CES2 is abundant in the small intestine [40]. Second, recombinant CES2 is subject to post-translational modifications specifically in insect cells, which somewhat affects the interactions between the protein and substrates/inhibitors. Thus, the differences between CES2 expressions in the human liver and intestine and recombinant factor may contribute to distinct inhibitory behaviors against HLM, HJM and recombinant CES2. Moreover, given the characteristics and site-specific expressions of CES1 and CES2, excipients that inhibit the CES2 activity may primarily affect the intestinal absorption of oral formulations, whereas excipients inhibiting the CES1 activity in the liver could alter drug elimination.

To compare the inhibitory effects of SLS, RH40, Tween 20 and EL 35 on human CES1A1 and CES2 activities, the IC_50_ values of the *p*-nitrophenyl acetate hydrolase activity were evaluated (Fig. 5). SLS and RH 40 inhibited CES1A1 more effectively than CES2. In contrast, Tween 20 and EL 35 were more effective against CES2. Moreover, SLS and Tween 20 showed strongest inhibition on CES1A1 (IC_50_ = 0.20 μg/ml) and CES2 (IC_50_ = 3.16 μg/ml), respectively. Collectively, the different inhibitory potentials may be ascribed to the structural differences between excipients and enzymes.

According to our results, SLS, RH40, Tween 20 and EL35 are all surfactants and exhibit the most potent inhibitory on human CES1A1 and CES2. Surfactants are commonly used as wetting agents, emulsifiers, solubilizers and carriers in new drug delivery systems to improve the dissolution and absorption of poorly soluble drugs [41]–[43]. The development of poorly soluble pharmaceutical therapies (Biopharmaceutics Classification System II and IV) has become increasingly common in the pharmaceutical industry [4]. As such, there may exist an increasing need for a wide variety of vehicle formulations such as surfactants that can enhance bioavailability of poorly soluble drugs. Despite their important role, recent studies have demonstrated that surfactants may have biological effects [2], [44]. For example, several surfactants can inhibit transporters proteins [8], [45] and can also impact on the enzyme activity. It is reported that Tween 20, EL35, S40 and F68 can inhibit CYP450 intensely [17].

In addition to its major role in the hydrolysis of numerous therapeutically active compounds, CES1 has been reported to be a key enzyme responsible for the biotransformation of a number of pyrethroid organophosphate and carbamate insecticides [36], [46]. CES1 activity is crucial to the detoxification process of these increasingly used insecticides. Thus, patients exposed to those surfactants may be prone to suffering from toxic reactions after accidental poisoning.

Our current study provides preliminary results that warrant further experiments. First, the study was limited to *in vitro* experiments. *In vitro* and *in vivo* findings are usually inconsistent, necessitating the need for corresponding experiments to be conducted *in vivo*. Second, the effect of individual excipients on CES activity was evaluated in this study. Most formulations include more than one excipient; thus, further studies with additional cautions are necessary when extending the combined effects of 2 or more excipients used together or with patients who are receiving more than 2 medications simultaneously, because such individual excipients probably act synergistically.

In summary, our current study represents the first investigation of potential inhibitory effects of 25 common excipients on human CES1A and CES2 and raises the awareness of the ability of surfactants to inhibit enzyme activity. Information related to pharmaceutical excipients with known potential side effects is important in clinical practice, particularly in patients suffering from disorders that can be aggravated by these excipients. The data presented in this study demonstrate that caution should be exercised during drug formulation and subsequent administration, and excipient-mediated repressive effects on CES merit further investigation.

## References

[1] Buggins TR, Dickinson PA, Taylor G (2007) The effects of pharmaceutical excipients on drug disposition. Adv Drug Deliv Rev 59: 1482–503. 18198495 10.1016/j.addr.2007.08.017

[2] Hirama S, Tatsuishi T, Iwase K, Nakao H, Umebayashi C, et al (2004) Flow-cytometric analysis on adverse effects of polysorbate 80 in rat thymocytes. Toxicology 199: 137–43. 15147788 10.1016/j.tox.2004.02.017

[3] Nahata MC (2009) Safety of “inert” additives or excipients in paediatric medicines. Arch Dis Child Fetal Neonatal Ed 94: F392–3. 19846397 10.1136/adc.2009.160192

[4] Thackaberry EA, Kopytek S, Sherratt P, Trouba K, McIntyre B (2010) Comprehensive investigation of hydroxypropyl methylcellulose, propylene glycol, polysorbate 80, and hydroxypropyl-beta-cyclodextrin for use in general toxicology studies. Toxicol Sci 117: 485–92. 20643750 10.1093/toxsci/kfq207

[5] Hamid KA, Katsumi H, Sakane T, Yamamoto A (2009) The effects of common solubilizing agents on the intestinal membrane barrier functions and membrane toxicity in rats. Int J Pharm 379: 100–8. 19555752 10.1016/j.ijpharm.2009.06.018

[6] EMA (2005) Reflection paper: formulations of choice for the paediatric population. EMA/CHMP/PEG/194810/2005.

[7] Ursino MG, Poluzzi E, Caramella C, De Ponti F (2011) Excipients in medicinal products used in gastroenterology as a possible cause of side effects. Regul Toxicol Pharmacol 60: 93–105. 21354240 10.1016/j.yrtph.2011.02.010

[8] Cornaire G, Woodley J, Hermann P, Cloarec A, Arellano C, et al (2004) Impact of excipients on the absorption of P-glycoprotein substrates in vitro and in vivo. Int J Pharm 278: 119–31. 15158955 10.1016/j.ijpharm.2004.03.001

[9] Lin Y, Shen Q, Katsumi H, Okada N, Fujita T, et al (2007) Effects of Labrasol and other pharmaceutical excipients on the intestinal transport and absorption of rhodamine123, a P-glycoprotein substrate, in rats. Biol Pharm Bull 30: 1301–7. 17603171 10.1248/bpb.30.1301

[10] Sachs-Barrable K, Thamboo A, Lee SD, Wasan KM (2007) Lipid excipients Peceol and Gelucire 44/14 decrease P-glycoprotein mediated efflux of rhodamine 123 partially due to modifying P-glycoprotein protein expression within Caco-2 cells. J Pharm Pharm Sci 10: 319–31. 17727795

[11] Shen Q, Li W, Lin Y, Katsumi H, Okada N, et al (2008) Modulating effect of polyethylene glycol on the intestinal transport and absorption of prednisolone, methylprednisolone and quinidine in rats by in-vitro and in-situ absorption studies. J Pharm Pharmacol 60: 1633–41. 19000368 10.1211/jpp/60.12.0009

[12] Shen Q, Lin Y, Handa T, Doi M, Sugie M, et al (2006) Modulation of intestinal P-glycoprotein function by polyethylene glycols and their derivatives by in vitro transport and in situ absorption studies. Int J Pharm 313: 49–56. 16500056 10.1016/j.ijpharm.2006.01.020

[13] Shono Y, Nishihara H, Matsuda Y, Furukawa S, Okada N, et al (2004) Modulation of intestinal P-glycoprotein function by cremophor EL and other surfactants by an in vitro diffusion chamber method using the isolated rat intestinal membranes. J Pharm Sci 93: 877–85. 14999725 10.1002/jps.20017

[14] Zhu S, Huang R, Hong M, Jiang Y, Hu Z, et al (2009) Effects of polyoxyethylene (40) stearate on the activity of P-glycoprotein and cytochrome P450. Eur J Pharm Sci 37: 573–80. 19442720 10.1016/j.ejps.2009.05.001

[15] Engel A, Oswald S, Siegmund W, Keiser M (2012) Pharmaceutical excipients influence the function of human uptake transporting proteins. Mol Pharm 9: 2577–81. 22808947 10.1021/mp3001815

[16] Bravo Gonzalez RC, Huwyler J, Boess F, Walter I, Bittner B (2004) In vitro investigation on the impact of the surface-active excipients Cremophor EL, Tween 80 and Solutol HS 15 on the metabolism of midazolam. Biopharm Drug Dispos 25: 37–49. 14716751 10.1002/bdd.383

[17] Ren X, Mao X, Cao L, Xue K, Si L, et al (2009) Nonionic surfactants are strong inhibitors of cytochrome P450 3A biotransformation activity in vitro and in vivo. Eur J Pharm Sci 36: 401–11. 19041719 10.1016/j.ejps.2008.11.002

[18] Imai T (2006) Human carboxylesterase isozymes: catalytic properties and rational drug design. Drug Metab Pharmacokinet 21: 173–85. 16858120 10.2133/dmpk.21.173

[19] Hatfield MJ, Potter PM (2011) Carboxylesterase inhibitors. Expert Opin Ther Pat 21: 1159–71. 21609191 10.1517/13543776.2011.586339 PMC3139797

[20] Satoh T, Taylor P, Bosron WF, Sanghani SP, Hosokawa M, et al (2002) Current progress on esterases: from molecular structure to function. Drug Metab Dispos 30: 488–93. 11950776 10.1124/dmd.30.5.488

[21] Fukami T, Takahashi S, Nakagawa N, Maruichi T, Nakajima M, et al (2010) In vitro evaluation of inhibitory effects of antidiabetic and antihyperlipidemic drugs on human carboxylesterase activities. Drug Metab Dispos 38: 2173–8. 20810539 10.1124/dmd.110.034454

[22] Xu YJ, Zhang CL, Li Xiping, Wu Tao, Ren XH, et al.. (2013) Evaluation of the inhibitory effects of antihypertensive drugs on human carboxylesterase in vitro. Drug Metab Pharmacokinet. 10.2133/dmpk.dmpk-12-rg-143 23648675

[23] Takahashi S, Katoh M, Saitoh T, Nakajima M, Yokoi T (2008) Allosteric kinetics of human carboxylesterase 1: species differences and interindividual variability. J Pharm Sci 97: 5434–45. 18383336 10.1002/jps.21376

[24] Humerickhouse R, Lohrbach K, Li L, Bosron WF, Dolan ME (2000) Characterization of CPT-11 hydrolysis by human liver carboxylesterase isoforms hCE-1 and hCE-2. Cancer Res 60: 1189–92. 10728672

[25] Zhang CL, Gao P, Yin WF, Xu YJ, Xiang DC, et al (2012) Dexamethasone regulates differential expression of carboxylesterase 1 and carboxylesterase 2 through activation of nuclear receptors. J Huazhong Univ Sci Technolog Med Sci 32: 798–805. 23271276 10.1007/s11596-012-1037-z

[26] Watanabe A, Fukami T, Nakajima M, Takamiya M, Aoki Y, et al (2009) Human arylacetamide deacetylase is a principal enzyme in flutamide hydrolysis. Drug Metab Dispos 37: 1513–20. 19339378 10.1124/dmd.109.026567

[27] Takahashi S, Katoh M, Saitoh T, Nakajima M, Yokoi T (2009) Different inhibitory effects in rat and human carboxylesterases. Drug Metab Dispos 37: 956–61. 19225040 10.1124/dmd.108.024331

[28] Golightly LK, Smolinske SS, Bennett ML, Sutherland EW 3rd, Rumack BH (1988) Pharmaceutical excipients. Adverse effects associated with inactive ingredients in drug products (Part I). Med Toxicol Adverse Drug Exp 3: 128–65. 3287089

[29] Tompkins L, Lynch C, Haidar S, Polli J, Wang H (2010) Effects of commonly used excipients on the expression of CYP3A4 in colon and liver cells. Pharm Res 27: 1703–12. 20503067 10.1007/s11095-010-0170-2 PMC3718039

[30] Johnston TP, Palmer WK (1993) Mechanism of poloxamer 407-induced hypertriglyceridemia in the rat. Biochem Pharmacol 46: 1037–42. 8216346 10.1016/0006-2952(93)90668-m

[31] Johnston TP, Palmer WK (1997) Effect of poloxamer 407 on the activity of microsomal 3-hydroxy-3-methylglutaryl CoA reductase in rats. J Cardiovasc Pharmacol 29: 580–5. 9213198 10.1097/00005344-199705000-00003

[32] Johnston TP, Baker JC, Hall D, Jamal S, Palmer WK, et al (2000) Regression of poloxamer 407-induced atherosclerotic lesions in C57BL/6 mice using atorvastatin. Atherosclerosis 149: 303–13. 10729380 10.1016/s0021-9150(99)00339-1

[33] Johnston TP, Baker JC, Jamal AS, Hall D, Emeson EE, et al (1999) Potential downregulation of HMG-CoA reductase after prolonged administration of P-407 in C57BL/6 mice. J Cardiovasc Pharmacol 34: 831–42. 10598127 10.1097/00005344-199912000-00010

[34] Blonder JM, Baird L, Fulfs JC, Rosenthal GJ (1999) Dose-dependent hyperlipidemia in rabbits following administration of poloxamer 407 gel. Life Sci 65: PL261–6. 10576602 10.1016/s0024-3205(99)00495-6

[35] Wasan KM, Subramanian R, Kwong M, Goldberg IJ, Wright T, et al (2003) Poloxamer 407-mediated alterations in the activities of enzymes regulating lipid metabolism in rats. J Pharm Pharm Sci 6: 189–97. 12935429

[36] Ross MK, Crow JA (2007) Human carboxylesterases and their role in xenobiotic and endobiotic metabolism. J Biochem Mol Toxicol 21: 187–96. 17936933 10.1002/jbt.20178

[37] Hosokawa M, Furihata T, Yaginuma Y, Yamamoto N, Koyano N, et al (2007) Genomic structure and transcriptional regulation of the rat, mouse, and human carboxylesterase genes. Drug Metab Rev 39: 1–15. 17364878 10.1080/03602530600952164

[38] Maruichi T, Fukami T, Nakajima M, Yokoi T (2010) Transcriptional regulation of human carboxylesterase 1A1 by nuclear factor-erythroid 2 related factor 2 (Nrf2). Biochem Pharmacol 79: 288–95. 19715681 10.1016/j.bcp.2009.08.019

[39] Godin SJ, Crow JA, Scollon EJ, Hughes MF, DeVito MJ, et al (2007) Identification of rat and human cytochrome p450 isoforms and a rat serum esterase that metabolize the pyrethroid insecticides deltamethrin and esfenvalerate. Drug Metab Dispos 35: 1664–71. 17576809 10.1124/dmd.107.015388

[40] Schwer H, Langmann T, Daig R, Becker A, Aslanidis C, et al (1997) Molecular cloning and characterization of a novel putative carboxylesterase, present in human intestine and liver. Biochem Biophys Res Commun 233: 117–20. 9144407 10.1006/bbrc.1997.6413

[41] Ahuja N, Katare OP, Singh B (2007) Studies on dissolution enhancement and mathematical modeling of drug release of a poorly water-soluble drug using water-soluble carriers. Eur J Pharm Biopharm 65: 26–38. 16962750 10.1016/j.ejpb.2006.07.007

[42] Sintov AC, Shapiro L (2004) New microemulsion vehicle facilitates percutaneous penetration in vitro and cutaneous drug bioavailability in vivo. J Control Release 95: 173–83. 14980766 10.1016/j.jconrel.2003.11.004

[43] Vandecruys R, Peeters J, Verreck G, Brewster ME (2007) Use of a screening method to determine excipients which optimize the extent and stability of supersaturated drug solutions and application of this system to solid formulation design. Int J Pharm 342: 168–75. 17573214 10.1016/j.ijpharm.2007.05.006

[44] Iwase K, Oyama Y, Tatsuishi T, Yamaguchi JY, Nishimura Y, et al (2004) Cremophor EL augments the cytotoxicity of hydrogen peroxide in lymphocytes dissociated from rat thymus glands. Toxicol Lett 154: 143–8. 15475188 10.1016/j.toxlet.2004.08.003

[45] Rege BD, Kao JP, Polli JE (2002) Effects of nonionic surfactants on membrane transporters in Caco-2 cell monolayers. Eur J Pharm Sci 16: 237–46. 12208453 10.1016/s0928-0987(02)00055-6

[46] Maxwell DM, Brecht KM (2001) Carboxylesterase: specificity and spontaneous reactivation of an endogenous scavenger for organophosphorus compounds. J Appl Toxicol 21 Suppl 1S103–7. 11920929 10.1002/jat.833

